# Inequity in health care utilization in Ecuador: an analysis of current issues and potential solutions

**DOI:** 10.1186/1475-9276-11-S1-A6

**Published:** 2012-01-23

**Authors:** Daniel Lopez-Cevallos, Chunhuei Chi

**Affiliations:** 1 Community Health, Western Oregon University, Monmouth, Oregon, 97361, USA; 2 International Health Program, Oregon State University, Corvallis, Oregon, 97331, USA

## Background

From an equity perspective, a health system needs to address social, political and economic inequalities that prevent people to achieve higher standards of living and exert their right to health [1,2]. The 2008 Ecuadorian Constitution guarantees the right to health, an important contributing factor to good living or *Sumak Kawsay *[3]*.* The purpose of this study was to analyze socioeconomic inequalities and its relationship to provision of health care services in Ecuador, and to discuss relevant policy alternatives.

## Methods

Our analysis on social and economic determinants of health care utilization used the 2004 National Demographic and Maternal & Child Health Survey (ENDEMAIN) as the main dataset, which included a representative national population sample. To estimate the effects of social and economic variables on health care utilization, we applied two types of analysis, multilevel multivariate analyses by using MLWiN 2.02, and spatial analyses by using GeoDa 0.9.5 [4].

## Results

We found that various layers of social inequalities persist in the Ecuadorian society. Indigenous, low-income, and rural households are particularly limited in their ability to access health care services in Ecuador. Additionally, we explored the influence of migration and remittances in reducing inequalities in health care utilization. We found that having a migrant in the household and receiving remittances improved use of antiparasitic medicines and to a lesser extent curative services, particularly among low-income Ecuadorians. Despite the drastic concentration in urban areas of private and public health care providers, we found that density of public practice health personnel is positively associated with use of preventive and curative care, particularly among rural households.

## Conclusions

Our study indicates that major social, economic, and geographic inequalities persist and are associated with access to health care services in Ecuador. Efforts to transform the health care system should go beyond its curative focus to consider an equitable distribution of health care resources and reducing financial and social barriers. These can be best achieved through strengthening government sector public health services, as our study suggest. Further, promoting family and community orientation of services [5,6], and strengthening spaces for public participation and control [7] are also essential to reduce inequalities in access to health care.
